# Effect of biomedical complications on very and extremely preterm children's language

**DOI:** 10.3389/fpsyg.2023.1163252

**Published:** 2023-06-28

**Authors:** Virginia Varela-Moraga, Benjamín Diethelm-Varela, Miguel Pérez-Pereira

**Affiliations:** ^1^Departamento de Fonoaudiología, Facultad de Medicina, Universidad de Chile, Santiago, Chile; ^2^Department of Molecular Genetics and Microbiology, School of Biological Sciences, Pontificia Universidad Católica de Chile, Santiago, Chile; ^3^Departamento de Psicoloxía Evolutiva e da Educación, Universidade de Santiago de Compostela, Santiago de Compostela, Spain

**Keywords:** biomedical complication, preterm children, language acquisition, parental questionnaires, psychological development

## Abstract

**Introduction:**

Very and extremely preterm children have been found to show delays in the development of language in early years. In some investigations, however, a rigorous control of biomedical complications, such as Periventricular Leukomalacia (PVL), Intraventricular Hemorrhage (IVH) or Bronchopulmonary Dysplasia (BPD), does not always exist. For that reason, a confounding effect of low gestational age and biomedical complications may lead to erroneous conclusions about the effect of gestational age.

**Methods:**

In this investigation we compare language development [use of words, sentence complexity and mean length of the three longest utterances (MLU3)] of three groups of Chilean children at 24 months of age (corrected age for preterm children). The first group was composed of 42 healthy full-term children (Full term group: FT), the second group of 60 preterm children born below 32 gestational weeks without medical complications (low risk preterm group: LRPT), and the third group was composed of 64 children below 32 gestational weeks who had medical complications (High risk preterm group: HRPT). The three groups were similar in terms of gender distribution, maternal education, and socio-economic environment. The instrument used to assess language was the Communicative Development Inventories (CDI). In addition, the Ages and Stages Questionnaire-3 (ASQ-3) was also used to assess other developmental dimensions.

**Results:**

The results indicate that HRPT and LRPT children obtained significantly lower results than the FT group in the three language measures obtained through the CDI. No significant differences were observed between the HRPT and the LRPT groups, although the HRPT obtained the lowest results in the three CDI measures. The results obtained through the administration of the ASQ-3 confirm the delay of both preterm groups in communicative development when compared to the FT group. No significant differences between the FT and the PT groups were observed in gross motor, fine motor and problem solving dimensions of the ASQ-3. The LRPT group obtained results that were significantly higher than those of the FT group and the HRPT group in gross motor development.

**Discussion:**

These results seem to indicate that the area of language development is particularly influenced by very or extremely low gestational age.

## Introduction

Premature children are considered a vulnerable population due to their immaturity as a result of their early birth. Very premature and extremely premature children, who were born with < 32 and 28 weeks of gestation, respectively (Goldenberg et al., 2008; Blencowe et al., 2019), present high morbidity and are exposed to greater biological risk. The lower the gestational age and the lower the birth weight, the more likely the presence of associated complications, chronic pathologies, and developmental delays (Bhutta et al., 2002). These biomedical complications could eventually have an impact on cognitive, linguistic, and behavioral performance during childhood. The probability of suffering cerebral palsy and neurosensory disorders increases as the gestational age decreases (Synnes et al., 1994). Children with gestational age (GA) between 31 and 23 weeks are those who have a higher probability of suffering neurodevelopmental disorders (Kilbride et al., 2004; Thorngren-Jerneck and Herbst, 2006; GuangXi Cooperative Research Group for Extremely Preterm Infants et al., 2019). GuangXi Cooperative Research Group for Extremely Preterm Infants et al. (2019) evaluated a sample of children born before 28 weeks of gestation and found that GA is a predictive factor for neurodevelopmental disorders, which means that as GA decreases, neurosensory disorders increase. Similarly, Baron et al. (2014) found that GA was the most important factor that determined differences in neuropsychological, intellectual, and behavioral functioning, and Anderson and Doyle (2008) found that 40% of the children under 26 weeks of gestation had cognitive delays.

Among the most frequent early biomedical complications found in preterm infants, bronchopulmonary dysplasia (BPD) stands out for its association with the risk of delay in cognitive and language development (Singer et al., 2001; Rvachew et al., 2005; Anderson and Doyle, 2006; Short et al., 2007; Sansavini et al., 2011; Gallini et al., 2021; Katz et al., 2022). The complications and sequelae caused by intraventricular hemorrhage (IVH) on cognitive, linguistic, and educational achievements are related to its severity, in such a way that the higher the grade of IVH, the worse its effects, with IVH of grades III and IV being particularly serious (Sherlock et al., 2005; Luu et al. al., 2009; Srinivasakumar et al., 2013; Vohr, 2022). A similar situation occurs with the presence of periventricular leukomalacia (PVL), which has a negative effect on cognitive and language development (Ohgi et al., 2005; Resic et al., 2008). Low Apgar scores in premature infants who manage to survive are related to the appearance of HIV, PVL, necrotizing enterocolitis, and retinopathy of prematurity, in addition to a long hospital stay (Phalen et al., 2012). Soares et al. (2017) found that the risk for language development in preterm children under 32 weeks of gestation was highly associated with the presence of intraventricular hemorrhage, bronchopulmonary dysplasia, maternal age of < 18 years, birth weight of < 1,000 g, and prolonged hospitalization (15–30 days minimum).

In addition to the aforementioned biomedical complications, there are environmental factors that provide additional influence (Reidy et al., 2013). Although neonatal medical risk consistently displays a negative impact on early childhood outcomes, socioeconomic and demographic risks (such as mothers who are single, of young age, or with less than a high school education) may affect cognitive, language, and motor delays, and the opposite, good socioeconomic status positively impacts on development (Mangin et al., 2016; Nyman et al., 2017; Kilbride et al., 2022).

When these biomedical complications occur in preterm newborns, the presence of negative consequences later in development is highly likely. Preterm children who have presented evident and demonstrable damage through medical examinations and procedures are considered at high risk for possible delays or disorders in their development. On the contrary, those who present few or no associated alterations are considered a low-risk group (da Ribeiro et al., 2017). In any case, there is so much heterogeneity among preterm children that even low-risk preterm infants show mixed outcomes in their language development (Casiro et al., 1990; Menyuk et al., 1995; Stolt et al., 2007; Cattani et al., 2010; Pérez-Pereira et al., 2014; Pérez-Pereira and Cruz, 2018; Suttora et al., 2020; Pérez-Pereira, 2021).

What appears repeatedly in research on very preterm and extremely preterm children with low birth weight and the presence of biomedical complications are low scores in language assessments. Most assessments of language development of children approximately 24 months of age have been carried out using the MacArthur-Bates scales or Communicative Development Inventories (CDIs) (Fenson et al., 2006). The spread of adaptations of this parental report instrument to many different languages has permitted the assessment of a large number of very young children. It would have otherwise been very difficult to assess these children through individual administration of conventional tests.

Most studies using the CDI have shown that very and extremely preterm children present lower scores and an evident delay in early lexical and morphosyntactic development or in vocabulary composition when compared to their term-born peers (Jansson-Verkasalo et al., 2004; Sansavini et al., 2006, 2011; Foster-Cohen et al., 2007; Gayraud and Kern, 2007; Stolt et al., 2007, 2009, 2012, 2017; Schults et al., 2013; Sentenac et al., 2020; Tulviste et al., 2020). The performance of preterm children is worse the lower the weight and gestational age, especially with < 32 weeks of gestational age (Foster-Cohen et al., 2007; Gayraud and Kern, 2007).

The Ages and Stages Questionnaire-3rd edition (ASQ-3) (Squires et al., 2009), another parental questionnaire, has also been used as a screening test of infant and child development in 5 areas: communication, gross motor, fine motor, problem-solving, and personal social. This questionnaire has been proposed as a useful screening instrument for the following up of preterm children as a population at risk of developmental delay (Skellern et al., 2001; Marks et al., 2009; Simard et al., 2012; Agarwal et al., 2016; Ballantyne et al., 2016; Al-Hindi et al., 2021). It has been found that the scores obtained by preterm children approximately 4 years of age in the five areas explored by the ASQ-3 decrease as the gestational age of the children decreases and, coherently, that the risk of developmental delay of preterm children between 25 and 40 weeks of gestation ascends as their gestational age descends. The percentage of 4-year-old children with rates of abnormal total problems scale in the ASQ ranges from 4.2% among term-born children to 37.5% among children born at 24–25 weeks' gestation (*p* < 0.001) in a large Dutch study with 1,439 preterm children and 544 FT children (Kerstjens et al., 2012). A similar pattern has been observed in all underlying ASQ domains. Coincident results were found by Hua et al. (2021) in a study carried out with a huge sample of 137,530 Chinese preschoolers between 3 and 5 years of age who ranged from very and moderately preterm (< 34 gestational weeks) to post-term (< 41 gestational weeks). The authors found that the mean scores obtained in the five domains assessed with the ASQ-3 increased as the GA of the children grew. There was, however, a decrease in the scores obtained by the children born post-term when compared to the term-born children (Hua et al., 2021). Coherently, the percentage of children at risk of developmental delay in the five ASQ-3 domains increased as gestational age decreased, with, again, the exception of post-term children who presented an increase in relation to the FT children. The adjusted risks of GAs (very and moderately preterm, late preterm, early term, and post-term groups) on suspected developmental delays were observed in communication (odds ratios (ORs) were 1.83, 1.28, 1.13, and 1.21, respectively, each *p* < 0.05), gross motor skill (ORs were 1.67, 1.38, 1.10, and 1.05, respectively, each *p* < 0.05), and personal-social behavior (ORs were 1.01, 1.36, 1.12, and 1.18, respectively, each *p* < 0.05) (Hua et al., 2021).

In a study with 52 infants, 12-month-old late preterm (GA of 34–36 weeks) and 156 full-term infants matched for sex, Ballantyne et al. (2016) observed a trend for late preterm infants to be at risk of communication and gross motor delays as measured through the ASQ-3 at 12 months of chronological age. Neonatal intensive care unit (NICU) admission has been found to increase the risk of developmental delay. In another comparative study with 44 late preterm and 44 full-term children, Gutiérrez Cruz et al. (2019) also observed that the late preterm infants had significantly lower scores (*p* < 0.005) in the dimension of communication of the ASQ-3.

Al-Hindi et al. (2021) administered the Saudi Arabian version of the ASQ-3 to a sample of 61 very preterm children (below 32 weeks) between 18 and 24 months of age. Twenty-six infants (42.6%) had at least one neurodevelopmental delay. The percentages found of children with developmental delays in the different dimensions were the following: communication skills (11.5%), gross motor (11.5%), fine motor (19.7%), problem-solving skills (19.7%), and personal-social skills (23%). Therefore, the domains of personal-social, problem-solving, and fine motor skills seem to be the most affected in Saudi Arabian infants.

Early age evaluations in preterm children are of great importance to identify children at risk of developmental delays and to implement intervention programs (Schults et al., 2013; Vohr, 2016). Parental report instruments such as the CDI or the ASQ-3 may be of great help in this regard.

The primary objective of this study was to compare the language development of three groups of Chilean children at 24 months of age. Three different measures of language development were obtained through the administration of the MacArthur-Bates scales: (1) use of words, (2) sentence complexity, and (3) mean length of the three longest utterances (MLU3). The three groups of children differed in terms of gestational age and in terms of the presence or not of additional biomedical complications: (1) healthy full-term children, (2) very and extremely preterm children with biomedical complications, and (3) very and extremely preterm children without serious biomedical complications. The research questions are as follows: (1) Do full-term children show better language development than the two groups of very and extremely preterm children? (2) Do the extremely and very preterm children with biomedical complications have worse language results than the very and extremely preterm children without biomedical complications?

A secondary aim was to compare other dimensions of psychological development (communication, social interaction, fine motor development, gross motor development, and problem-solving) among the three groups of participants. The research questions are as follows: (1) Do the full-term children show better performance in the five dimensions of psychological development than the two groups of extremely and very preterm children? (2) Do the preterm children with biomedical complications have worse results in the five dimensions of psychological development than the preterm children without biomedical complications?

The hypotheses of the study are as follows:

- Full-term (FT) children will outperform the two groups of very and extremely preterm children in the three language measures.
- High-risk very preterm and extremely preterm infants (HRPT) will perform worse and have a higher incidence of delay in the three measures of language development than the low-risk very and extremely preterm (LRPT) children as well as the full-term children.
- Full-term children will outperform the two groups of preterm children in the other developmental measures obtained through the ASQ-3 (communication, gross motor, fine motor, problem-solving, and personal-social development).
- High-risk very preterm and extremely preterm infants (HRPT) will perform worse and have a higher incidence of delay in the ASQ-3 five developmental measures than the other two groups (low-risk very and extremely preterm (LRPT) and full-term children).

## Method

### Participants

Three groups of Chilean children were studied at 24 months of age (corrected age for preterm children).

The first group was composed of 42 healthy full-term children (Full-term group: FT), born between 37 and 40 weeks of gestation, and without medical problems. The children were recruited in different preschool centers located in Santiago de Chile, and they were of similar gender distribution and socioeconomic and maternal education levels as the children from the two preterm groups. The FT children obtained an Apgar score at 1 min of 7 points or higher.

The second group consisted of 60 preterm children born below 32 gestational weeks without serious medical complications (low-risk preterm group: LRPT). The children attended a follow-up program for preterm children in the Hospital Roberto del Río and the health center Cordillera Oriente, both of them located in Santiago de Chile. The criteria to include the children in this group were that they had no serious neurological impairment (IVH of grades III or IV, PVL) or lung disease (BPD), that they had an Apgar score in the 1st min of 7 or above, and that they stayed in the neonatal intensive care unit (NICU) of the hospital for < 30 days after being born.

The third group was composed of 64 children below 32 gestational weeks who had medical complications (high-risk preterm group: HRPT). The children attended a follow-up program for preterm children in the Hospital Roberto del Río and the Health Center Cordillera Oriente, both located in Santiago de Chile. The children of this group were included if they had suffered any of the following biomedical conditions: serious neurological impairment (IVH of grades III or IV, PVL), bronchopulmonary dysplasia, Apgar score in the 1st min below 7 points, or a stay in the neonatal intensive care unit (NICU) of the hospital of 30 days or longer after being born.

All the children were assessed at 24 months of age (corrected age for the preterm children).

Table 1 displays descriptive data of the three groups.

**Table 1:** Descriptive data of the three groups: Mean and (SD)/frequency (%).

	**HRPT**	**LRPT**	**FT**
N	64	60	42
Gestational age	28.27 (2.03)	30.42 (0.81)	38.98 (0.95)
Apgar score	5.55 (2.42)	7.71 (0.73)	8.52 (0.67)
Days in NICU	32.42 (31.48)	6.98 (6.65)	0.0 (0.0)
Birth weight	1,139 (363)	1,503 (327)	3,317 (466)
Biomedical complications^*^	2.45 (1.11)	0 (0)	0 (0)
Gender (girl)	30 (47%)	33 (55%)	23 (55%)

^*^Number of biomedical complications.

Table 2 shows the educational level of the mothers per group. The following categorization was used:

**Table 2:** Mothers' educational level per group (frequency).

	**HRPT**	**LRPT**	**FT**
**Level**	**64**	**60**	**42**
0	1 (1,6%)	0 (0,0%)	0 (0,0%)
1	0 (0,0%)	1 (1,7%)	0 (0,0%)
2	4 (6,3%)	1 (1,7%)	1 (2,4%)
3	2 (3,1%)	6 (10,0%)	2 (4,8%)
4	28 (43,8%)	14 (23,3%)	10 (23,8%)
5	8 (12,5%)	7 (11,7%)	7 (16,7%)
6	8 (12,5%)	19 (31,7%)	11 (26,2%)
7	5 (7,8%)	5 (8,3%)	3 (7,1%)
8	8 (12,5%)	7 (11,7%)	8 (19,0%)

Level 0: no formal education; Level 1: basic or incomplete primary education; Level 2: complete basic or primary education; Level 3: middle or incomplete secondary education; Level 4: middle or complete secondary education; Level 5: incomplete technical studies; Level 6: complete technical studies; Level 7: incomplete university studies; and Level 8: complete university studies.

The X^2^ test revealed that there were no significant differences between groups regarding gender distribution (X^2^ = 1.015; df = 2.2; *p* = 0.602).

The X^2^ test indicates that there were no significant differences between groups regarding the educational level of the mothers (X^2^ = 20.012, df = 16.1, *p* = 0.220).

Therefore, the three groups could be considered comparable in relation to gender distribution and mother's educational level.

No children with cerebral palsy, metabolic or genetic syndromes, serious motor or sensorial problems, or pervasive developmental delay were admitted to the study.

### Instruments

The Mexican Spanish version of the Communicative Development Inventories (CDIs) (*Inventario para el Desarrollo de Habilidades Comunicativas:* IDHC) (Jackson-Maldonado et al., 2003) was used to assess the linguistic development of the participants. Given the age of the children, the form *Palabras y Enunciados* (Words and Sentences) for children between 16 and 30 months of age was given to the mothers. The CDI is a parental report instrument that has two parts. The first part, *Uso de las Palabras*, has two sections: (A) *List of words*, with a list of 680 words, organized into 23 categories, from which the parents must mark those that are produced by their child, with one point given for each marked word (out of the 680 words that make up the Mexican Spanish version, 666 (98%) have exactly the same form in Chilean Spanish, and 14 words have variations; the person in charge of the application explained their meanings to the parents when necessary); and (B) *Cómo usa y comprende el lenguaje el niño* (How children use and understand language), which contains five items that assess whether the child talks about situations to refer to the past, present, and future or with respect to absent objects or people and their search. The maximum score for this section is 5 points.

The second part of the Mexican Spanish CDI, *Oraciones y Gramática* (grammar and sentences), has three sections. The first one, A. *Formas de verbos* (Verb forms), explores the capacity of the child to produce forms of verbs in present (12 items), past (6 items), and imperative (6 items) for the three verb conjugations existing in Spanish. The maximum possible score is 24. The second section, B. *Combinación de palabras* (Word combination), asks the parents whether their child already combines words or not. If the answer is yes, the parents must give three examples of the longest utterances their child has recently produced. The mean length of these three longest utterances (MLU3) in words is obtained. If the child does not yet produce word combinations, the parents should stop filling out the inventory. The third part, C. *Complejidad de frases* (Sentence complexity), consists of 37 pairs of phrases. Both phrases express the same idea, although the second is always more complex (and evolved) from a morphosyntactic point of view. The parents are asked to mark the form that is more similar to that used by their child. One point is given if the parents mark the second option. The maximum possible score is 37 points.

For the purposes of the present research, only the scores obtained in the list of words produced (named *use of words*), MLU3, and sentence complexity were considered.

The Mexican Spanish CDI has good validity and reliability values (Thal et al., 2000; Jackson-Maldonado et al., 2003).

The Spanish version of the Ages and Stages Questionnaire-3 (ASQ-3) (Squires et al., 2009) was used to assess the psychological development of the participants. The form for age 24 months was used. The ASQ-3 is a parental questionnaire that assesses five areas of development: communication, gross motor, fine motor, problem-solving, and personal social. Each area contains six items assessing different abilities in each domain, which can be scored as 0 (not yet), 5 (sometimes), or 10 (yes). The maximum score for each dimension is 60 points. The score is interpreted according to a normative chart that is included in the questionnaire. According to this chart and the User's Guide, each child can be classified in one of three areas: (1) above the cutoff, when the child's development appears to be on schedule; (2) close to the cutoff, when it is necessary to provide learning activities and monitor; or (3) below the cutoff, when further assessment with a professional may be needed. The validity and reliability of the ASQ-3 reach good values (validity is 0.82 to 0.88, test–retest reliability is 0.91, and inter-rater reliability is 0.92) (Squires et al., 2015). The results obtained through the ASQ-3 are coincident with those obtained through the Bayley III scales (Agarwal et al., 2016; Mackin et al., 2017).

Structured interview. An interview adapted from that used by Pérez-Pereira et al. (2014) explored aspects such as socioeconomic indicators, parental educational level, family composition, health issues of the child and the parents, daily routines, and family history of language problems.

In addition, information on biomedical problems, gestational history, and delivery was also obtained from the records at the hospitals.

### Procedure

Approval of the *Comité de Ética para Investigaciones en Seres Humanos* (Ethics Committee for Research with Human Beings) of the Faculty of Medicine of the University of Chile was obtained to carry out this research (resolution No. 1026). Prior informed consent was also given by the participants' parents.

The preterm children were selected from the two previously reported medical centers: Hospital Roberto del Río and the Health Center Cordillera Oriente in Santiago de Chile, where the preterm children were included in a follow-up program. Information about the eligible children was obtained from these centers, and those who fulfilled the age requirements and the rest of the inclusion criteria previously specified were included.

The full-term participants that presented the age requirements and inclusion criteria were chosen from preschool centers in the north area of Santiago de Chile.

During the second semester of 2019, the instruments were administered in person. From the year 2020, because of the COVID-19 pandemic, the instruments were administered remotely (through email, SMS, WhatsApp, or video call). Previously, the mother (in most cases) or the father of each child was called by phone to arrange the modality of contact. In this first call, information on the study and instructions on how to proceed were given to the parents.

The first author carried out the assessment. The assessment took place when the children were 24 months old (±15 days), using the chronological age for the full-term children and the corrected age for the preterm children.

The parents filled out the two questionnaires (Mexican Spanish CDI and ASQ-3) and the structured interview.

### Analysis performed

Analyses of variance (ANOVA) were performed to compare the scores obtained by the three groups of children (FT, LRPT, and HRPT) in the three measures taken from the CDI—that is, use of words or word production, MLU3, and sentence complexity—and the five measures obtained through the ASQ-3. The SPSS-28 was used for the analyses.

In addition, and solely for discussion purposes, Pearson's correlations between the three language measures (IDHC) and the five measures of psychological development (ASQ-3) were obtained.

## Results

Table 3 shows the results obtained in the three measures of the IDHC taken under consideration.

**Table 3:** ANOVA comparisons among groups in the scores of the CDI [mean and (SD)].

	**HRPT**	**LRPT**	**FT**	**F**	**df**	***p***	**η^2^**
**N**	**64**	**60**	**42**				
Use of words	57.27 (63.44)	71.93 (63.16)	233.14 (167.71)	44.688	2.163	< 0.001^***^	0.354
MLU3	1.51 (0.77)	1.69 (0.69)	2.62 (1.21)	21.884	2.163	< 0.001^***^	0.212
Sentence complexity	0.81 (1.76)	0.92 (1.09)	7.50 (9.46)	28.618	2.163	< 0.001^***^	0.260

^***^p < 0.001.

As can be observed, the ANOVA results indicate that there are significant differences among the groups in the use of words, MLU3, or sentence complexity. A *post hoc* Bonferroni test (*p* < 0.05) reveals that those differences are due to the significantly higher results obtained by the FT children in relation to those obtained by the HRPT and the LRPT groups; there are no significant differences, however, between the HRPT and the LRPT groups in the use of words, MLU3, or sentence complexity. The relatively higher results obtained by the LRPT children in comparison with the HRPT children do not reach significance.

The results obtained in the ASQ-3 are shown in Table 4, together with the results of the ANOVA.

**Table 4:** ANOVA comparisons among groups in the scores of the ASQ-3 [mean and (SD)].

	**HRPT**	**LRPT**	**FT**	**F**	**df**	***p***	**η^2^**
Communication	28.52 (17.13)	33.00 (17.42)	48.69 (13.88)	18.785	2.163	< 0.001^***^	0.187
Gross motor	47.50 (13.09)	55.42 (5.92)	50.95 (8.78)	5.935	2.163	0.003^**^	0.068
Fine motor	45.78 (11.52)	48.17 (7.42)	47.38 (9.64)	0.573	2.163	0.565	0.007
Problem-solving	43.28 (13.66)	47.67 (12.12)	43.45 (10.03)	1.100	2.163	0.335	0.013
Personal social	42.42 (14.36)	47.00 (9.62)	49.64 (31.71)	5.403	2.163	0.005^**^	0.062

^***^p < 0.001. ^**^p < 0.01.

The area of *communication* is where the differences between preterm children and full-term children are the highest. The difference between the FT group, on the one hand, and the HRPT and the LRPT groups, on the other hand, is highly significant, and the effect size (η^2^) reaches nearly 19%. The Bonferroni *post hoc* test confirms that the difference found in the ANOVA is due to the significantly higher result obtained by the FT children in relation to both the HRPT and the LRFT children (*p* < 0.001).

In the areas of *fine motor* development and *problem-solving* capacity, there are no significant differences among the three groups, although, in this case, the group with the best results is the LRPT and not the FT group.

In the area of *gross motor* development, the LRPT group obtains the highest results, but now the ANOVA value reaches significance. The Bonferroni *post hoc* test indicates that there are significant differences between the LRPT and the HRPT groups and between the LRPT and FT groups (*p* < 0.001).

Finally, the ANOVA results indicate that there are significant differences between the groups in the area of *personal-social* development. The *post hoc* Bonferroni test confirms that the FT group obtains significantly higher results than the HRPT (*p* < 0.001) and that the LRPT group obtains significantly higher results than the HRPT group. The difference between the FT and the LRPT groups does not reach significance.

The two groups of PT children also differed in birth weight and gestational age. In order to check whether these differences would have affected the results, we have introduced these variables as covariates in univariate general linear models. Dependent variables use of words, MLU3, and sentence complexity (obtained through the CDI) and communication, gross motor, fine motor, problem-solving, and personal social (obtained through the ASQ-3) were successively introduced. Belonging to the LRPT or HRPT group has been used as an independent variable (fixed factor). Birth weight and gestational age were introduced as covariates. The results show that gestational age does not affect any of the results, while birth weight only affects gross motor skills and personal-social development. The pattern of results regarding the independent variable (LRPT vs. HRPT group) does not change in relation to the results of the ANOVA test reported in Tables 3, 4, with the sole exception of personal-social development, which when introducing the covariates does not show a significant difference between the LRPT and HRPT groups (F = 1.532, *p* = 0.218).

Considering the interpretation of the mean scores of each group in the different ASQ-3 areas, the area of *communication* is clear, that is, in which the two groups of preterm children obtain the worse results. In relation to the limit score in this area (which is 25.17), the FT children are clearly above the cutoff, while the HRPT and the LRPT children are close to the cutoff and would need monitoring and additional learning activities to help their development. In the four remaining areas, the scores obtained by the three groups are above the cutoff, with the children showing age-appropriate performance.

Spearman's correlations are shown in Table 5. The results clearly show that the correlations between the ASQ-3 communication scores and the IDHC scores (use of words r = 0.735, MLU3 r = 0.800, and sentence complexity = 0.511) are the highest (*p* < 0.001).

**Table 5:** Pearson's correlations (bilateral) between IDHC and ASQ-3 measures.

	**Communication**	**Gross motor**	**Fine motor**	**Problem-solving**	**Personal social**
Use of words	0.735^***^	0.112	0.166^*^	0.119	0.371^***^
MLU3	0.800^***^	0.126	0.186^*^	0.178^*^	0.373^***^
Sentence complexity	0.511^***^	0.029	0.138	0.078	0.250^**^

^*^p < 0.05. ^**^p < 0.01. ^***^p < 0.001.

## Discussion

In relation to the first objective of the study, the FT group obtained significantly higher scores in the use of words, MLU3, and sentence complexity than the other two PT groups: HRPT and LRPT, as Table 3 shows. These results agree with those found in other studies comparing FT and very and extremely preterm children through the CDI (Jansson-Verkasalo et al., 2004; Sansavini et al., 2006, 2011; Foster-Cohen et al., 2007; Gayraud and Kern, 2007; Stolt et al., 2007, 2009, 2012, 2017; Schults et al., 2013; Sentenac et al., 2020; Tulviste et al., 2020). Therefore, we can say that hypothesis 1 is confirmed. The effect size (η^2^) found in the use of words (0.354) was higher than the effect size obtained in the two morphosyntactic measures: MLU3 (0.212) and sentence complexity (0.260). This is probably because morphosyntactic development is barely developed at the age of 24 months, and, logically, the differences found are lower than in lexical development.

The second hypothesis is only partially confirmed; however, because even though the differences between the HRPT group and the FT group are clearly significant in the three language measures, the differences between the HRPT and the LRPT groups do not reach significance. These results do not match those found in other studies, which point to a negative effect of biomedical complications on language development (Singer et al., 2001; Ohgi et al., 2005; Rvachew et al., 2005; Sherlock et al., 2005; Anderson and Doyle, 2006; Short et al., 2007; Resic et al., 2008; Luu et al., 2009; Sansavini et al., 2011; Phalen et al., 2012; Srinivasakumar et al., 2013; Soares et al., 2017; Gallini et al., 2021; Katz et al., 2022; Vohr, 2022). Few exceptions to this widespread pattern are available. Although the children from the LRPT group obtained higher mean scores than the children from the HRPT group in the use of words (71.93 vs. 57.27), MLU3 (1.69 vs. 1.51), and sentence complexity (0.92 vs. 0.81, respectively), these differences are not significant. Probably, the reduced number of participants in the sample makes it difficult to find significant differences. Furthermore, we have used the criteria of counting the number of risk circumstances (from 1 to 5), and the combinations of these biomedical risks could vary. We have not analyzed the effect of singular risk conditions (such as having BPD or IVH of grades III and IV) because the number of children who suffered from them was rather limited. In addition, many children from the HRPT group presented comorbidities, and two or more biomedical risks were present.

The comparison of the results obtained by the three groups in the five areas of psychological development assessed by the ASQ-3 indicates that hypothesis 3 is only partially confirmed. The area of *communication* is the only one in which the FT group obtains significantly higher results than the two groups of very and extremely PT children (see Table 4). This result agrees with those found by other studies (Ballantyne et al., 2016; Gutiérrez Cruz et al., 2019; Hua et al., 2021), which point to a higher risk of delay of PT children in communication. In this area, the effect size (η^2^) is higher (0.187).

Along the same line, the results obtained in *personal-social* development seem to support the hypothesis that FT children should have higher results than the LRPT and the HRPT groups. The FT group obtained the highest results, followed by the LRPT group, and finally the HRPT group. In this case, significant differences were found between the FT and the HRPT groups but not between the FT and the LRPT groups. This result is, for the most part, coincident with those found by other studies (Al-Hindi et al., 2021; Hua et al., 2021), although the mean scores obtained by all the groups are above the cutoff.

In the area of *fine motor development*, however, there were no significant differences among the groups, and hypothesis 3 has not been supported. The group of LRPT children even obtained the highest scores in this dimension, although no significant differences were found. This result does not agree with that found by Al-Hindi et al. (2021), who found a relatively high percentage (19.7%) of very preterm children who showed developmental delays in fine motor development. In any case, the mean scores of all the groups are appropriate to their age.

Similar results were found for *problem-solving*. Although the group of LRPT children obtained the highest results, no significant differences were found among the groups. This result is not in agreement with what has been found in other studies (Kerstjens et al., 2012; Al-Hindi et al., 2021). Again, the mean scores of the three groups were above the cutoff, and no developmental risk has been found.

Finally, the results observed in *gross motor* development are in contradiction with hypothesis 3 because the LRPT group obtained results that were significantly higher than those of the FT group and, even more so, than those of the HRPT group. In any case, no group seemed to be at risk of developmental delay in this area, since the mean scores of all the groups were in the normal range, according to ASQ-3 norms. The results obtained in gross motor development in our study are dissonant with those studies that found that PT children obtained significantly lower results than FT children (Ballantyne et al., 2016; Hua et al., 2021).

In relation to hypothesis 4, as expected, the HRPT group obtained lower results than the FT group in the five areas of development explored by the ASQ-3, although these differences reach significance in only communicative and personal-social development. The HRPT group also obtained lower results than the LRPT group in all the areas explored, although those differences reach significance only in gross motor development.

Clearly, communicative development seems to be the area most affected by prematurity. The development of both the HRPT and LRPT children in the remaining areas seems to unfold according to expectations. Curiously, LRPT children have even higher results than the FT children in gross motor, fine motor, and problem-solving development, although only in gross motor development do differences reach significance.

Therefore, the results obtained with the ASQ-3 do not support the idea that very and extremely preterm children show delays in all areas of development at 24 months of age, as suggested by other studies carried out with 4-year-old children (Kerstjens et al., 2012; Hua et al., 2021). Their development seems to follow normal patterns in gross motor, fine motor, problems solving, and personal-social development, particularly if these PT children do not have biomedical complications. The improvement in the care provided to PT children in the hospitals in the last years might be also related to the results found.

The high correlations found between the results of communication in the ASQ-3 and the use of words MLU3 and sentence complexity in the CDI reinforce the idea that language and communication are particularly affected in very and extremely preterm children regardless of whether they present medical complications or not.

## Conclusion

The extremely and very preterm children studied obtained significantly lower results than the full-term children in all language and communicative measures taken at 24 months of age. Contrary to expectations, the biomedical risk factors have not shown any significant effect on the development of language and communication, although HRPT children obtained the lowest results in all measures.

The development of other areas is not so much affected by preterm birth and LRPT, and HRPT children seem to develop according to expectations. LRPT children obtain higher results than the HRPT and even the FT children in certain areas of development. This indicates that biomedical complications seem to have a detrimental effect on development, particularly gross motor, fine motor, problem-solving, and personal-social development.

One limitation of the present research is the relatively reduced number of participants in the study. It is possible that differences between the HRPT and the LRPT groups could appear, particularly in language and communication, if the number of participants was larger. In the same vein, the singular effect of different biomedical complications (including necrotizing enterocolitis) could be studied with a larger number of participants.

One strength of the present research is that two groups of very and extremely preterm children, with and without biomedical complications, were studied.

## Data availability statement

The raw data supporting the conclusions of this article will be made available by the authors, without undue reservation.

## Ethics statement

The studies involving human participants were reviewed and approved by Comité de Ética de Investigación en Seres Humanos (CEISH) Facultad de Medicina Universidad de Chile. Written informed consent to participate in this study was provided by the participants' legal guardian/next of kin.

## Author contributions

VV-M contributed to conception and design of the study and performed assessments of all study participants. BD-V organized the database and performed the statistical analysis. MP-P contributed to the conception and design of the study and the analysis and interpretation of the results by critically reviewing it and wrote the draft of the manuscript. All authors contributed to manuscript review, read, and approved the submitted version.

## Editor's note

Maria-José Ezeizabarrena edited the article in collaboration with Melita Kovacevic, University of Zagreb, Zagreb, Croatia.
